# Supplementary material for: Detection of prions from spiked and free-ranging carnivore feces
Source: Sci Rep. 2024 Feb 15;14:3804. doi: 10.1038/s41598-023-44167-7 (PMC10869337; doi:10.1038/s41598-023-44167-7)
Supplement: Supplementary file 1 — Supplementary Information. [file 41598_2023_44167_MOESM1_ESM.pdf]

SUPPLEMENTARY INFORMATION

Detection of prions from spiked and free-ranging carnivore feces

H. N. Inzalaco[a], E. E. Brandell[a], S.P. Wilson[b], M. Hunsaker[a], D. R. Stahler[c], K. Woelfel[d], D. P. Walsh[e], T. Nordeen[b], D. J. Storm[f], S. S. Lichtenberg[g], W. C. Turner[h]

[a]Wisconsin Cooperative Wildlife Research Unit, Department of Forest and Wildlife Ecology, University of Wisconsin, Madison, Madison, WI 53706, USA

[b]Nebraska Game and Parks Commission 2200 N 33rd St. P.O. Box 30370, Lincoln, NE 68503

[c]Yellowstone Center for Resources, Yellowstone National Park, WY 82190

[d]Wild and Free Wildlife Rehabilitation Program, 27264 MN-18 Garrison, MN 56450, USA

[e]U.S. Geological Survey, Montana Cooperative Wildlife Research Unit, University of Montana, Missoula, MT, USA

[f]Wisconsin Department of Natural Resources, Eau Claire, WI 54701 USA

[g]Department of Veterinary and Biomedical Sciences, University of Minnesota, St. Paul, MN 55108, USA

[h]U.S. Geological Survey, Wisconsin Cooperative Wildlife Research Unit, Department of Forest and Wildlife Ecology, University of Wisconsin – Madison, Madison, WI 53706, USA.

*Correspondence: inzalaco@wisc.edu

TABLES AND FIGURES

Supplementary Table S1. Ordered differences of the Least Squares mean amyloid formation rates between all spiked feces and chronic wasting disease (CWD)-positive brain homogenate used for spiking fecal samples. Statistical significance was determined by Tukey HSD multiple comparisons test.

Feces type	Feces type comparison	Difference	Pooled standard error	Lower CL	Upper CL	p-value
Raven	Fox	0.031677	0.003655	0.020052	0.043301	0.00E+00
Raven	Cougar	0.030095	0.003655	0.018471	0.04172	0.00E+00
Raven	Raccoon	0.011151	0.003655	-0.00047	0.022775	7.25E-02
Raven	Coyote	0.010118	0.003655	-0.00151	0.021743	1.51E-01
Raven	Bear	0.008386	0.003655	-0.00324	0.020011	3.95E-01
Raven	Wolf	0.003546	0.003655	-0.00808	0.015171	9.94E-01
Cougar	Fox	0.001582	0.003655	-0.01004	0.013206	1.00E+00
Wolf	Fox	0.028131	0.003655	0.016506	0.039755	0.00E+00
Wolf	Cougar	0.026549	0.003655	0.014925	0.038174	2.22E-11
Wolf	Raccoon	0.007605	0.003655	-0.00402	0.019229	5.42E-01
Wolf	Coyote	0.006572	0.003655	-0.00505	0.018197	7.36E-01
Wolf	Bear	0.00484	0.003655	-0.00678	0.016465	9.48E-01
Wolf	Eagle	0.021598	0.003655	0.009973	0.033222	3.17E-07
Eagle	Fox	0.049729	0.003655	0.038104	0.061353	0.00E+00
Eagle	Cougar	0.048147	0.003655	0.036523	0.059772	0.00E+00
Eagle	Raven	0.018052	0.003655	0.006427	0.029676	4.94E-05
Eagle	Raccoon	0.029202	0.003655	0.017578	0.040827	0.00E+00
Eagle	Coyote	0.02817	0.003655	0.016545	0.039794	0.00E+00
Eagle	Bear	0.026438	0.003655	0.014813	0.038062	4.05E-11
Bear	Fox	0.023291	0.003655	0.011666	0.034915	2.18E-08
Bear	Cougar	0.021709	0.003655	0.010085	0.033334	2.67E-07
Bear	Raccoon	0.002765	0.003655	-0.00886	0.014389	9.99E-01
Bear	Coyote	0.001732	0.003655	-0.00989	0.013357	1.00E+00
Raccoon	Fox	0.020526	0.003655	0.008902	0.032151	1.58E-06
Raccoon	Cougar	0.018945	0.003655	0.00732	0.030569	1.49E-05
Crow	Fox	0.065923	0.003655	0.054299	0.077548	0.00E+00
Crow	Cougar	0.064342	0.003655	0.052717	0.075966	0.00E+00
Crow	Raven	0.034246	0.003655	0.022622	0.045871	0.00E+00
Crow	Raccoon	0.045397	0.003655	0.033773	0.057022	0.00E+00
Crow	Coyote	0.044365	0.003655	0.03274	0.055989	0.00E+00
Crow	Bear	0.042633	0.003655	0.031008	0.054257	0.00E+00
Crow	Wolf	0.037792	0.003655	0.026168	0.049417	0.00E+00
Crow	Eagle	0.016195	0.003655	0.00457	0.027819	5.03E-04
Coyote	Fox	0.021559	0.003655	0.009934	0.033183	3.36E-07

Coyote	Cougar	0.019977	0.003655	0.008353	0.031602	3.50E-06
Coyote	Raccoon	0.001033	0.003655	-0.01059	0.012657	1.00E+00
Brain	Fox	0.107407	0.003655	0.095782	0.119031	0.00E+00
Brain	Cougar	0.105825	0.003655	0.094201	0.11745	0.00E+00
Brain	Raven	0.07573	0.003655	0.064105	0.087354	0.00E+00
Brain	Raccoon	0.086881	0.003655	0.075256	0.098505	0.00E+00
Brain	Coyote	0.085848	0.003655	0.074223	0.097472	0.00E+00
Brain	Bear	0.084116	0.003655	0.072491	0.09574	0.00E+00
Brain	Wolf	0.079276	0.003655	0.067651	0.0909	0.00E+00
Brain	Eagle	0.057678	0.003655	0.046054	0.069303	0.00E+00
Brain	Crow	0.041483	0.003655	0.029859	0.053108	0.00E+00

Supplementary Table S2. Percent recovery of seeding material based on amyloid formation rate (AFR) values of 10-fold dilution spikes in fecal samples for each species to AFR values of 10-fold dilutions of the reference sample.

Species	Spike dilutions and percent recovery based on AFR					
	10^6	10^5	10^4	10^3	100	10
Crow	96%	94%	78%	45%	0%	0%
Eagle	62%	59%	57%	62%	43%	0%
Wolf	68%	47%	32%	33%	44%	60%
Raven	66%	45%	43%	37%	0%	0%
Bear	50%	42%	38%	15%	10%	0%
Coyote	68%	42%	27%	3%	8%	0%
Raccoon	55%	48%	29%	12%	0%	0%
Cougar	39%	15%	7%	8%	0%	0%
Fox	36%	12%	5%	5%	0%	13%

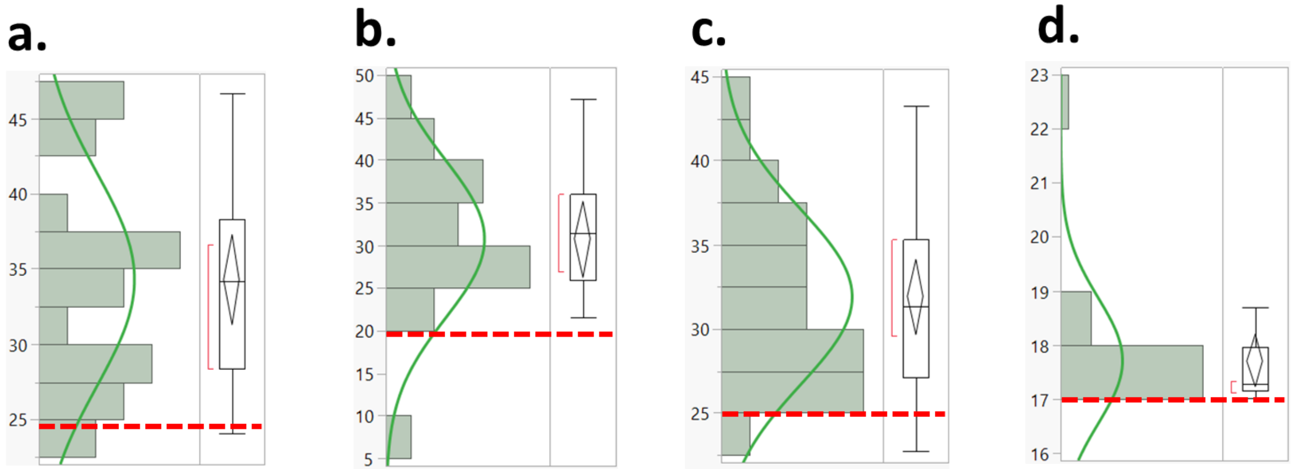

Supplementary Figure S1 Histogram plots depicting empirical distributions of threshold times for 24 technical replicates of unspiked feces from **(a)** bear (34.3, ±7.1), **(b)** crow (M,30.7; SD, 9.2), **(c)** eagle (M, 31.9; SD, 5.2), and **(d)** raccoon (M, 17.7; SD, 1.2). Horizontal dotted red lines indicate cycle end-times that excluded ≥95% of the false seeding that occurred for each of these feces.

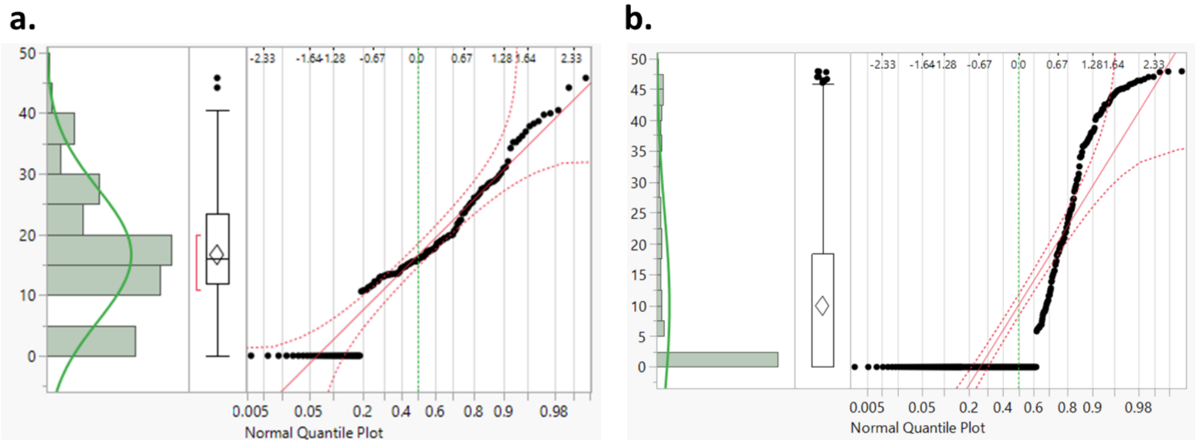

Supplementary Figure S2. Non-normal distributions of the time (hours)-to-threshold data for predator and scavenger fecal samples. Histogram and normal quantile plot depicting distribution of hours to threshold for (a) coyote fecal sample data depicted in "Figure 3" (N, 168; M, 16.6; SD, 10.9; Goodness of Fit P, < .0001) and (b) cougar fecal sample data depicted in "Figure 4" (N, 384; M, 9.9; SD, 15.27, Goodness of Fit P, < .0001)
